# PERMA-Profiler as a multidimensional measure of well-being in a French context

**DOI:** 10.1038/s41598-026-57354-z

**Published:** 2026-06-12

**Authors:** Jourdan Julie, Serrand Chris, Chevallier Thierry, Schandrin Aurélie, Stéphane Raffard

**Affiliations:** 1 Department of Adult Psychiatry, Nîmes University Hospital, 585 Chemin du Mas de Lauze, 30900 Nîmes, France; 2 EPSYLON Laboratory UR4556, Department of Psychology, University of Montpellier Paul Valéry, Montpellier, France; 3 Department of Biostatistics, Epidemiology, Public Health and Innovation in Methodology, CHU Nîmes, Nîmes, France; 4 University Department of Adult Psychiatry, CHU Montpellier, Montpellier, France

**Keywords:** Wellbeing, PERMA, French translation and validation, Factor analysis, Psychometric properties, Psychology, Quality of life

## Abstract

Subjective well-being is a central component of physical and mental health and is increasingly recognized as a key indicator of quality of life. Despite the widespread use of the PERMA framework, no validated French version of the PERMA-Profiler is currently available. The aim of this study was to translate and culturally adapt the PERMA-Profiler into French and to examine its psychometric properties in a large sample of adults. A total of 612 French-speaking adults completed the French version of the PERMA-Profiler as well as measures of flourishing, anxiety and depression. The sample was randomly divided into two independent subsamples to conduct exploratory factor analysis (EFA) and confirmatory factor analysis (CFA). The EFA suggested a parsimonious three-factor solution, while comparative model tests confirmed the theoretical five-factor structure of the PERMA model. The CFA conducted on an independent subsample strongly confirmed a correlated five-factor model, as well as a second-order hierarchical model reflecting a general well-being factor. The PERMA-Profiler demonstrated good to excellent internal consistency for most dimensions and for the total score, although the “engagement” dimension showed lower reliability, which is consistent with previous validation studies. Test–retest analyses indicated good temporal stability for the total score and most subscales. Convergent validity was confirmed by strong positive associations with flourishing and perceived happiness. Discriminant validity was evidenced by weaker associations with anxiety, depression, loneliness and negative emotions than with flourishing, although some correlations with HADS anxiety and depression scores reached moderate to strong magnitude. Overall, the French version of the PERMA-Profiler has robust psychometric properties and supports the multidimensional structure of psychological well-being proposed by the PERMA framework. This instrument is a reliable and theoretically grounded tool for assessing the well-being of French-speaking adult population, while highlighting the distinct psychometric behavior of the Engagement dimension.

**Supplementary Information:**

The online version contains supplementary material available at 10.1038/s41598-026-57354-z.

## Introduction

Subjective well-being (SWB) refers to people’s cognitive and affective evaluations of their own lives, including reflective judgments such as life satisfaction and the balance between positive and negative emotions^1^. Establishing this conceptual basis is essential for understanding the consistent associations observed between SWB and physical and mental health outcomes. Many studies have shown that higher well-being is associated with better physical health, both in the general population^2^ and in people suffering from chronic somatic diseases such as cardiovascular diseases^3^, rheumatoid arthritis^4^, diabetes^5^ or HIV^6^. Increased well-being is also related to greater life satisfaction^7^, more effective stress management^8^, lower risk of mood disorders and reduced morbidity^8^ and fewer physical symptoms with greater pain tolerance^9,10^. SWB is therefore considered a reliable and sensitive indicator of overall quality of life^11–13^.

Although there is no universal consensus on the precise definition of SWB, two major theoretical traditions dominate the field. The hedonic tradition defines well-being as the pursuit of pleasure, enjoyment and the absence of psychological distress^14,15^. The eudaimonic tradition emphasizes meaning, purpose, personal growth, virtue and self-actualization, reflecting a deeper assessment of whether one is living in accordance with one’s values and potential^16,17^. Ryff’s model of psychological well-being (PWB) exemplifies this eudaimonic tradition through six dimensions: autonomy, personal growth, self-acceptance, environmental mastery, life purpose, and positive relationships^16^. In contrast, the SWB tradition focuses on cognitive evaluations and affective balance such as life satisfaction^18,19^. Although these models emphasize different facets of well-being, they are empirically correlated and conceptually complementary, which has led several authors to propose integrated frameworks combining hedonic and eudemonic perspectives^20–22^.

Among these integrative models, Seligman’s PERMA framework^23^ is one of the most influential. He conceptualizes well-being as comprising five measurable components: positive emotions (P), engagement (E), positive relationships (R), meaning (M), and accomplishment (A). Positive Emotion refers to feelings such as pleasure, joy and satisfaction^24^. Engagement corresponds to the psychological state of flow, characterized by intense absorption and concentration in an activity^24^. Relationships include feelings of support, love and connection with others^23^. Meaning reflects having a purpose and belonging to something larger than oneself^24,25^. Accomplishment refers to achieving goals, demonstrating skills and effectively fulfilling daily responsibilities^25^. Collectively, these components represent a multidimensional but coherent model of well-being that integrates both hedonic and eudaimonic dimensions.

The PERMA model has attracted growing interest in both research and practical applications, leading to the development of several measurement tools^26–28^. Among these, the PERMA-Profiler^24^ is the most widely used self-assessment questionnaire for evaluating the five components of the PERMA model. It comprises 23 items, including 15 main items assessing positive emotions, engagement, relationships, meaning, and accomplishment, and eight additional items assessing physical health, negative emotions, loneliness, and perceived happiness^29^. In the original English validation, the PERMA-Profiler demonstrated acceptable internal consistency, test–retest reliability and a factor structure largely consistent with the theorical model^24^. However, subsequent validation studies have yielded inconsistent results across cultural contexts. For example, in Australia, neither the original five-factor model nor a unidimensional structure provided a satisfactory fit^30^. In contrast, validation studies conducted in Turkey^31^ and Italy^32^ supported the original five-factor structure proposed by Seligman^23^. These inconsistencies suggest that the factor structure of the PERMA-Profiler may be influenced by cultural differences, linguistic factors, sample characteristics (e.g., age distribution, overrepresentation of students), or methodological variations. To date, no validated French version of the PERMA-Profiler is available.

Given the conceptual importance of assessing both the hedonic and eudaimonic dimensions of well-being, as well as the inconsistencies reported in previous psychometric studies, this study had two main objectives: (1) to translate and culturally adapt the PERMA-Profiler into French using a rigorous multi-step procedure; and (2) to examine its psychometric properties, including factor structure, internal consistency, test–retest reliability and convergent and discriminant validity in a large sample of French adults.

In line with previous work, we expected the French version of the PERMA-Profiler to reproduce a multidimensional structure reflecting the theoretical components of the PERMA framework, while recognizing that alternative three- or four-factor structures reported in the literature could also emerge^24,32,33^. We anticipated that each dimension would exhibit satisfactory internal consistency and acceptable temporal stability^24,32^. With regard to conceptual validity, we expected strong positive associations between the PERMA dimensions and flourishing, as both constructs share substantial theoretical overlap within the eudaimonic tradition of well-being. Flourishing reflects optimal psychological functioning, including meaning, purpose, positive relationships, self-esteem and optimism^34,35^, which correspond closely to several core components of the PERMA framework. Previous research has demonstrated strong positive associations between PERMA scores and flourishing measures, supporting their conceptual convergence while maintaining their distinct operationalization^24^. In particular, flourishing has been widely used as an external benchmark in PERMA-Profiler validation studies, providing a theoretically grounded criterion for assessing convergent validity^24^. In contrast, we expected PERMA scores to show weaker and negative associations with indicators of psychological distress such as anxiety and depression, consistent with the theoretical distinction between well-being and psychopathology^36,37^. We also expected negative associations with loneliness and negative emotions and positive associations with perceived health, although these variables were treated as complementary indicators rather than independent external validation measures. These indicators correspond to additional single-item components included in the PERMA-Profiler. They are commonly examined separately in validation studies to further characterize the well-being/unwell-being continuum, rather than being treated as independent external measures^24,28^. In addition, perceived happiness (assessed through a single item included in the PERMA-Profiler) was expected to be strongly positively associated with PERMA scores. However, as this indicator is part of the PERMA-Profiler itself and not an independent external measure, these associations were considered descriptive and were not interpreted as formal tests of convergent validity. Finally, based on previous research, we expected small associations between PERMA scores and sociodemographic variables. Age was expected to show weak associations with well-being, while the direction of this association was considered uncertain given mixed findings in the literature. Gender differences were expected to be small in magnitude. Given these inconsistencies, these effects were examined in an exploratory manner^38–40^.

## Method

### Participants

Participants were recruited between March and September 2023 through two complementary procedures: (1) online dissemination of the study on social media (Facebook, LinkedIn and Instagram) and (2) face-to-face recruitment conducted in a quiet office in the Department of Adult Psychiatry at Nîmes University Hospital. Participants were encouraged to share the study with their family, friends, students, colleagues and other social contacts in order to obtain a heterogeneous sample representative of the general French population.

Socio-demographic information included age, gender, level of education, employment status, nationality and mother tongue. Participants also indicated whether they had a history of psychiatric diagnosis or were currently taking psychotropic medication. Exclusion criteria were: (a) being under 18 years of age; (b) not having sufficient command of French; and (c) having a reported history of traumatic brain injury. Participation was voluntary and uncompensated.

All procedures were conducted in accordance with ethical standards and applicable regulations. The study protocol was approved by the Northwest III Ethics Committee (Caen University Hospital) under number 2023-A00666-39. This study was pre-registered on ClinicalTrials.gov (ID: NCT05930353). All participants provided informed consent.

### Procedure

After receiving written and verbal information about the study, participants completed a series of questionnaires either online or during a face-to-face session, depending on their preference. In both cases, identical questionnaire content and administration procedures were used. Participants first completed the PERMA Profiler, followed by the additional measures. They were invited to ask questions before and after the session.

To assess test–retest reliability, all participants who had agreed to be recontacted at the end of the T1 assessment were invited to complete the questionnaire again 30–38 days after the first administration (by email for online participants and by phone for those recruited face-to-face, consistent with their initial mode of participation). The same series of questionnaires was administered at time 2 (T2). Data were anonymized after collection and data entry.

Each questionnaire (T1 and T2) included an attention control item. Data screening and exclusion criteria are described in the Data Preparation section.

### Translation and adaptation into the French cultural context

First, we received permission from Margaret Kern, the creator of the PERMA Profiler, to adapt the questionnaire in France via her website. The translation process followed a structured seven-step methodology based on Vallerand^41^, involving forward translation, back-translation and expert review.

Step 1 involved five bilingual translators (with backgrounds in psychology and psychometric testing) independently translating the original English items into French. A scientific committee (SC) compared the versions and produced a preliminary synthetic version. This version was reviewed by an expert panel and validated as a first working version. Step 2 consisted of face-to-face comprehension testing with 10 participants representative of the French general population. Their feedback helped identify unclear formulations and improve item clarity. Step 3 incorporated participants’ feedback into a revised version of the questionnaire, which was then reexamined and approved by the expert panel. Step 4 included a second round of comprehension testing with 10 new participants, conducted by the principal investigator (JJ). Minor refinements were made, leading to a final version (VF). Step 5 involved back-translation of the VF by three native English speakers who were unfamiliar with the original questionnaire. Step 6 compared the original version and the back-translation to verify conceptual equivalence between the two English versions. Step 7 finalized the process by submitting the French version and its back-translation to the expert panel for final validation. No major discrepancies were found between the original and translated versions.

### Measures

The PERMA-Profiler^24^ was the primary instrument under investigation. It contains 23 items, including 15 core items assessing the five PERMA dimensions (Positive Emotion, Engagement, Relationships, Meaning and Accomplishment) rated on an 11-point scale from 0 (“never”) to 10 (“always” or completely agree”). The instrument also included additional single-item indicators (physical health, negative emotions, loneliness, and perceived happiness), which are not included in the computation of the PERMA total score. Higher scores indicate greater well-being. In the present sample, internal consistency indices for the PERMA dimensions are reported in the Results section.

To assess convergent validity, participants completed the Flourishing Scale^34,42^, an 8-item self-report measure of global psychological well-being encompassing meaning, relationships, self-esteem and optimism. This instrument was selected because its eudemonic content conceptually overlaps with several PERMA dimensions and strong positive correlations were expected. In the present sample, the Flourishing Scale showed good internal consistency (Cronbach’s α = 0.89; McDonald’s ω = 0.89).

To assess discriminant validity, we used the Hospital Anxiety and Depression Scale (HADS)^43^ which assesses symptoms of anxiety and depression and is theoretically distinct from well-being. Internal consistency was satisfactory for both the anxiety (α = 0.80; ω = 0.81) and depression (α = 0.75; ω = 0.77) subscales in the present sample. In the present study, these additional PERMA items were analyzed separately as internal complementary indicators of the well-being/unwell-being continuum. Importantly, they were not treated as independent external validation measures, but rather as descriptive extensions of the PERMA construct. As single-item measures, internal consistency coefficients are not applicable for these indicators.

### Data availability

The authors confirm that all studies were carried out in compliance with current guidelines and regulations. French PERMA-Profiler scale is available free of charge on the Open Science Framework (OSF) and can be used by clinicians and scientists alike. All data has been made publicly available on OSF (https://osf.io/53t8c).

### Data preparation

The initial dataset included 612 adults who responded to the T1 (time 1) assessment. Educational attainment was recorded for all participants, including a specific category of “university students”, who represented 5.1% of the sample. University students are often overrepresented in psychological research, particularly in studies relying on online recruitment^44–46^, and because young adults tend to report higher levels of subjective well-being than older adults^38–40^, we examined the proportion of students to ensure that it did not skew the sample. In the present study, the percentage of students closely matched French national estimates^47^, and the entire sample was retained.

Data cleaning was performed in accordance with established psychometric recommendations^48,49^. Participants were excluded from psychometric analyses when they had missing values on any of the fifteen core PERMA items, in accordance with official scoring guidelines^24^. For secondary instruments (Flourishing Scale; HADS), missing data were handled by pairwise deletion, in accordance with their validated scoring procedures^34,43^. Each questionnaire included an attention control item, and participants who failed this item or who provided repetitive responses (e.g., identical responses) were excluded in accordance with methodological recommendations for data quality assurance^50^. All data were anonymized immediately after collection.

For factor analysis purposes, the complete T1 data set was randomly divided into two independent subsamples of 306 participants each. The first subsample (G1) was used for exploratory factor analysis, and the second (G2) for confirmatory factor analysis (CFA). The two groups did not differ significantly in terms of age, gender, educational level, psychiatric history, or use of psychotropic medications. The mode of administration (online or face-to-face) differed between subsamples and reflected participants’ preferred modality of questionnaire completion rather than systematic assignment. Identical materials and procedures were used across administration modes and no statistical adjustment for this variable was applied in subsequent analyses. A subgroup of participants from the initial T1 sample was invited to complete the T2 assessment approximately 30–38 days later, either online or by telephone, consistent with their initial mode of participation. All participants who had agreed to be recontacted at T1 and for whom sufficient contact information was available were invited to participate. This subgroup therefore constitutes a convenience subsample rather than a systematically selected group. Retest data were carefully checked for duplicate identifiers and inconsistencies. After removing three duplicate entries in T1 and excluding cases with missing PERMA values, the final matched T1–T2 sample comprised 175–179 individuals depending on the dimension analyzed and was used exclusively for test–retest reliability analyses. The number of participants included in each analysis therefore ranged from 175 to 179 depending on missing data for specific dimensions.

Prior to modeling, univariate distributions were examined using descriptive indices (mean, standard deviation, skewness, kurtosis), calculated using the psych software package^51^. The skewness and kurtosis values for the PERMA items and subscales remained within the recommended limits for robust maximum likelihood estimation (|skewness|< 2 and |kurtosis|< 7)^52^. Multivariate outliers were identified using Mahalanobis distances on thirteen continuous indicators. Thresholds based on χ^2^ distributions with *p* = 0.99 and *p* = 0.999^53^ identified 26 and 10 extreme cases, respectively. Since removing these observations did not significantly alter factor loadings, reliability indices, or correlation patterns, all cases were retained, in accordance with current recommendations for robustness and reproducibility^54^.

### Statistical analyses

All statistical analyses were performed using R (Version 4.4)^55^. The significance threshold was set at α ≤ 0.05 for all confirmatory analyses.

Prior to psychometric modeling, distributional properties and data screening procedures were examined as described in the Data Preparation section. Although variables showed only limited deviations from univariate normality^49,52^, PERMA items were treated as ordered categorical variables in the main confirmatory analyses, in accordance with methodological recommendations for Likert-type scales^56^.

Exploratory factor analysis (EFA) and confirmatory factor analysis (CFA) were performed on two independent subsamples, as described in the Data Preparation section. EFA was performed using principal axis factorization with promax rotation, in accordance with best practices for correlated psychological constructs^57^. The number of factors was determined by parallel analysis and examination of the scree plot. Sampling adequacy and factorability were assessed using the Kaiser–Meyer–Olkin index and Bartlett’s sphericity test. The CFA was performed using the lavaan software package^58^. Given the ordinal nature of the items, the primary CFA models were estimated using the WLSMV estimator. To assess the robustness of the results, sensitivity analyses were also performed by treating the items as continuous and using the robust maximum likelihood estimator (MLR). Model fit was evaluated using the χ^2^ statistic, the Comparative Fit Index (CFI), the Tucker–Lewis Index (TLI), the Root Mean Square Error of Approximation (RMSEA) with its 90% confidence interval, and the Standardized Root Mean Square Residual (SRMR). Model fit was interpreted according to the criteria proposed by Hu and Bentler^59^ and widely accepted psychometric guidelines.

Internal consistency was assessed using Cronbach’s alpha and McDonald’s total omega coefficient^60^. Omega coefficients were estimated based on the correlated 5-factor CFA model as omega provides a more accurate estimate of reliability in multidimensional constructs compared to alpha, particularly when the assumption of tau-equivalence is not met^61,62^. The hierarchical omega coefficient was not computed, as no bifactor model was retained as the primary measurement model. Test–retest reliability was examined using intraclass correlation coefficients (ICC, two-way random effects, absolute agreement), following the recommendations of McGraw and Wong^63^ and the interpretation guidelines of Koo & Li^64^.

Convergent and discriminant validity were examined using Pearson correlations between PERMA scores, the Flourishing Scale and HADS anxiety and depression scores. Correlations were calculated using pairwise deletion to maximize available information, in accordance with the scoring recommendations of Diener et al.^34^ and Zigmond and Snaith^43^. Given the primarily confirmatory nature of the main psychometric analyses and the limited number of theoretically driven tests, no correction for multiple comparisons was applied. This choice is consistent with recommendations suggesting that strict corrections (e.g., Bonferroni) may increase the risk of Type II errors and are not systematically required in theory-driven analyses^65,66^. In line with common practices in psychometric validation studies, emphasis was therefore placed on the magnitude, direction, and theoretical coherence of associations rather than solely on adjusted p-values.

Finally, multiple linear regressions were conducted to examine whether PERMA-Profiler scores (total score and dimensions) predicted scores on external well-being and distress measures (Flourishing Scale, HADS anxiety and depression), while adjusting for sociodemographic variables (age, gender, education). These models were specified to evaluate the extent to which PERMA scores accounted for variance in external criteria within a multivariate framework. Linear modeling assumptions were verified by inspecting residual distributions, Q-Q plots, homoscedasticity, and influence diagnostics. No influential observations exceeded standard Cook’s distance thresholds.

## Results

### Translation and adaptation of the French version

During the finalization of the French version of the PERMA-Profiler, the supervisory committee was unable to reach immediate consensus on three items (A1, M2 and P2) for which two competing formulations seemed conceptually acceptable. In order to document the adaptation process transparently, both versions of each item were subjected to a back-translation procedure. No major semantic differences were identified during this stage and the final selection of formulations was made on the basis of the convergence of expert judgments with an emphasis on clarity and conceptual proximity to the original version. The final French version of the PERMA-Profiler is presented in online Appendix A.

### Sample characteristics and descriptive statistics

The total sample comprised 612 adults aged between 18 and 86. From this group, two sub-samples of equal size (G1 and G2) were randomly selected for EFA and CFA.

Descriptive statistics for the composite dimensions of the PERMA-Profiler, the overall PERMA well-being score, the Flourishing Scale (FS), and the HADS anxiety and depression scores are presented in Table 1. The mean scores for the five dimensions of the PERMA model were above the midpoint of the scale (0–10), with averages ranging from 6.47 (Positive Emotions) to 7.06 (Accomplishment). The PERMA overall well-being score was 6.86 (SE = 1.37). The mean scores for negative emotions (M = 4.64; SD = 1.84) and loneliness (M = 3.52; SD = 2.94) reflected moderate levels of perceived distress, while the flourishing score (FS total) was relatively high (M = 44.00; SD = 8.22). The mean scores for anxiety (M = 8.11; SD = 3.95) and depression (M = 4.47; SD = 3.29) indicated low to moderate levels of symptomatology overall.

**Table 1:** Descriptive statistics for the PERMA-Profiler dimensions, the Flourishing Scale (FS), and HADS scores (N = 612).

Variable	Mean	SD	Min	Max	Skew	Kurt
PERMA–Positive emotions	6.47	1.66	1.00	9.67	− 0.67	0.15
PERMA–Engagement	6.95	1.46	2.00	10.00	− 0.51	0.21
PERMA–Relationships	7.05	1.77	0.00	10.00	− 0.77	0.81
PERMA–Meaning	6.75	1.95	0.00	10.00	− 0.80	0.56
PERMA–Accomplishment	7.06	1.42	0.67	10.00	− 1.00	1.69
PERMA–Negative emotions	4.64	1.84	0.00	10.00	0.18	− 0.79
PERMA–Health	7.17	1.92	0.00	10.00	− 0.95	1.00
PERMA–Loneliness	3.52	2.94	0.00	10.00	0.56	− 1.07
PERMA–Perceived Happiness	6.75	2.30	0.00	10.00	− 0.81	− 0.08
PERMA–Overall well-being	6.86	1.37	1.33	10.00	− 0.67	0.29
Flourishing Scale	44.00	8.22	13.00	56.00	− 0.62	− 0.14
HADS–Anxiety	8.11	3.95	0.00	20.00	0.18	− 0.64
HADS–Depression	4.47	3.29	0.00	16.00	0.66	− 0.25

All PERMA dimensions are measured on a scale of 0–10; the Flourishing Scale score on a scale of 8–56; HADS scores on a scale of 0–21. SD = standard deviation; Min = minimum; Max = maximum; Skew = skewness; Kurt = kurtosis.

The coefficients of asymmetry and kurtosis remained moderate overall (|asymmetry|≤ 1.05; |kurtosis|≤ 1.69), suggesting a limited deviation from the assumptions of univariate normality. Detailed descriptions for all dimensions (mean, standard deviation, minimum, maximum, skewness, kurtosis) are reported in Table 1. The distributions at the element level and the diagnostic normality figures (QQ plots, residuals) are provided in online Appendix B in order to meet methodological transparency requirements.

### Comparability of subsamples G1 and G2

The 612 participants who completed the PERMA Profiler at time T1 were randomly divided into two subsamples of equal size: G1 (n = 306), used for the EFA, and G2 (n = 306), used for the CFA. To verify that this random assignment did not introduce systematic bias, comparisons were conducted on sociodemographic and psychological variables (see Tables 2 and 3). No significant differences were observed between subsamples G1 and G2 and effect sizes were negligible across all variables. These results indicate that the two subsamples were comparable, supporting the validity of conducting exploratory and confirmatory factor analyses on independent samples.

**Table 2:** Comparison of continuous variables between subsamples G1 (EFA) and G2 (CFA).

Variable	G1 n	G1 M (ET)	G2 n	G2 M (ET)	t(df)	*p*	g [IC 95%]
Age	301	36.98 (14.50)	305	36.65 (14.86)	0.28 (604.0)	0.782	0.02 [− 0.14; 0.18]
PERMA–Positive emotions	306	6.51 (1.63)	306	6.43 (1.70)	0.57 (609.0)	0.571	0.05 [− 0.11; 0.20]
PERMA–Engagement	306	6.92 (1.54)	306	6.97 (1.39)	− 0.39 (608.0)	0.699	− 0.03 [− 0.19; 0.13]
PERMA–Relationships	306	7.01 (1.78)	306	7.10 (1.73)	− 0.68 (610.0)	0.498	− 0.05 [− 0.21; 0.10]
PERMA–Meaning	306	6.81 (1.92)	306	6.68 (1.95)	0.79 (610.0)	0.432	0.06 [− 0.09; 0.22]
PERMA–Accomplishment	306	7.08 (1.45)	306	7.04 (1.38)	0.37 (609.0)	0.711	0.03 [− 0.13; 0.19]
PERMA–Negative emotions	306	4.65 (1.86)	306	4.62 (1.81)	0.19 (609.0)	0.850	0.02 [− 0.14; 0.17]
PERMA–Health	306	6.75 (1.84)	306	6.58 (1.90)	1.13 (608.0)	0.257	0.09 [− 0.07; 0.25]
PERMA–Loneliness	306	3.48 (2.94)	306	3.57 (2.94)	− 0.38 (610.0)	0.701	− 0.03 [− 0.19; 0.13]
PERMA–Perceived Happiness	306	6.89 (2.10)	306	6.99 (2.13)	− 0.59 (610.0)	0.556	− 0.05 [− 0.21; 0.11]
PERMA–Overall well-being	306	6.87 (1.34)	306	6.86 (1.39)	0.11 (610.0)	0.913	0.01 [− 0.15; 0.17]
Flourishing scale	293	44.01 (8.08)	292	43.99 (8.37)	0.03 (582.0)	0.976	0.00 [− 0.16; 0.16]
HADS–Anxiety	299	8.01 (3.94)	296	8.21 (3.98)	− 0.61 (593.0)	0.539	− 0.05 [− 0.21; 0.11]
HADS–Depression	298	4.37 (3.30)	296	4.57 (3.27)	− 0.76 (592.0)	0.447	− 0.06 [− 0.22; 0.10]

Values are means (standard deviations). T-tests are Welch’s t-tests (unequal variances). G = Hedges’ g, with a 95% confidence interval. No test exceeds the significance threshold α = 0.05.

**Table 3:** Distribution of sociodemographic variables between subsamples G1 (EFA) and G2 (CFA).

Variable	Modality	G1 n (%)	G2 n (%)	χ^2^(df)	p	V [IC 95%]
Genre	Other	2 (0.7%)	4 (1.3%)	2.27(2)	0.321	0.02 [0.00; 1.00]
	Woman	218 (72.4%)	233 (76.4%)			
	Man	81 (26.9%)	68 (22.3%)			
Education	Primary school	1 (0.3%)	0 (0.0%)	9.19(6)	0.163	0.07 [0.00 ; 1.00]
	Secondary school	1 (0.3%)	6 (2.0%)			
	High school	51 (16.9%)	70 (23.0%)			
	Bachelors	70 (23.3%)	57 (18.7%)			
	University student	17 (5.6%)	14 (4.6%)			
	Masters	126 (41.9%)	123 (40.3%)			
	Doctorate	35 (11.6%)	35 (11.5%)			

The values are percentages. The χ^2^ tests evaluate the association between group membership (G1 vs. G2) and each categorical variable. V = Cramer’s V, with a 95% confidence interval. No test exceeds the significance threshold α = 0.05; the V values indicate associations of negligible magnitude.

### Exploratory factor analysis

An EFA was conducted on the 15 items of the PERMA Profiler in the G1 subsample (N = 306). Preliminary diagnostics show excellent data adequacy: the overall KMO index is very high (0.92), with individual measures ranging from 0.74 to 0.95, indicating sufficient homogeneity of correlations. Bartlett’s sphericity test is significant (χ^2^(105) = 2725.79, *p* < 0.001), confirming that the correlation matrix is not an identity matrix and is therefore factorizable.

A parallel analysis (100 iterations) was performed to determine the optimal number of factors. The observed eigenvalues exceeded the simulated values up to the third factor, suggesting a 3-dimensional structure. The scree plot also showed a clear inflection after the third factor, supporting this empirical solution. Although these results pointed toward a 3-factor structure, additional models were examined to account for theoretical considerations. In particular, the 5-factor model corresponding to the PERMA framework was retained for comparison, given its theoretical relevance. Three exploratory models were therefore estimated and compared: a 1-factor model, a 3-factor model (empirically suggested solution) and a 5-factor model (theoretical structure of PERMA). The extracted factor eigenvalues (SS loadings) for the 5-factor solution were 2.51, 2.33, 1.86, 1.79, and 0.99, respectively. Together, these factors accounted for 63.2% of the total variance, with individual contributions of 16.8%, 15.5%, 12.4%, 12.0%, and 6.6%, respectively.

The comparative results are presented in Table 4. The 1-factor model shows insufficient fit (RMSEA = 0.128; TLI = 0.80), suggesting that the items do not reflect a single underlying construct. The 3-factor model shows a more satisfactory fit (RMSEA = 0.093; TLI = 0.89; RMSR = 0.04), with a notable improvement in explained variance. The 5-factor model shows the best overall indices (RMSEA = 0.070; TLI = 0.94; RMSR = 0.021) and the highest proportion of explained variance, although the Bayesian Information Criterion slightly favored the three-factor solution, reflecting a trade-off between model fit and parsimony. Although the 3-factor solution showed acceptable global fit indices and was supported by parallel analysis, closer inspection of the factor structure revealed important interpretability limitations. Specifically, the third factor was weakly defined, with only one item (E3) showing a salient loading, while other items (P1 and P2) displayed substantial cross-loadings across multiple factors. This pattern suggests that the third factor does not represent a coherent or theoretically meaningful latent construct. Therefore, despite its relative parsimony, the 3-factor model was not retained for confirmatory testing, as it did not meet standard criteria for interpretability in latent variable modeling. In contrast, the 5-factor solution provided both better overall fit and strong theoretical coherence with the PERMA framework and was therefore retained for subsequent confirmatory analyses.

**Table 4:** Comparison of exploratory factor models (1, 3, and 5 factors).

	1-factor	3-factor	5-factor
RMSR	0.071	0.036	0.021
RMSEA [90% CI]	0.128 [0.118; 0.138]	0.093 [.081; 0.106]	0.070 [0.053; 0.088]
TLI	0.80	0.89	0.94
BIC	23.62	− 130.21	− 128.28
Explained variance	46.2%	56.0%	63.2%

Extraction by principal axes, promax rotation. RMSR = Root Mean Square of Residuals: measures the average size of off-diagonal residuals; lower values indicate a better fit of the model to the observed correlation matrix. RMSEA = Root Mean Square Error of Approximation: approximation error index that takes into account the complexity of the model; values < 0.08 indicate an acceptable fit, and < .06 a good fit. The 90% CI provides the confidence interval around the RMSEA. TLI = Tucker–Lewis Index: index comparing the fit of the model to that of a null model; values > 0.90 indicate a satisfactory fit. BIC = Bayesian Information Criterion: criterion penalizing model complexity; lower values reflect a better compromise between fit and parsimony. The explained variance corresponds to the proportion of total common variance explained by all factors.

The standardized factor loadings, communalities and uniquenesses of the 3-factor model are summarized in Table 5. The first factor mainly includes items related to meaning, accomplishment and engagement. The second factor corresponds to items in the Relationships dimension. The third factor groups together items related to positive emotions (P1–P2) as well as item E3, which has low commonality. The factors are moderately to strongly correlated (Φ = 0.36–0.65), suggesting substantial interdependence among the components of well-being. Importantly, adequacy in terms of global fit and residual structure does not necessarily imply conceptual interpretability. In the present case, the third factor appears to reflect shared variance across heterogeneous items rather than a distinct psychological dimension, further supporting its exclusion from confirmatory analyses.

**Table 5:** Factor loads (pattern matrix), commonalities, and uniquenesses for the 3-factor model.

Item	F1	F2	F3	h^2^	u^2^
PERMA P3	**0.55**	0.22	0.15	0.65	0.35
PERMA E1	**0.51**	− 0.10	0.26	0.41	0.59
PERMA E2	**0.62**	0.05	0.15	0.56	0.44
PERMA M1	**0.85**	0.12	− 0.17	0.71	0.29
PERMA M2	**0.97**	0.16	− 0.45	0.82	0.18
PERMA M3	**0.87**	0.14	− 0.21	0.76	0.24
PERMA A1	**0.78**	− 0.08	0.01	0.54	0.46
PERMA A2	**0.60**	− 0.09	0.16	0.43	0.57
PERMA A3	**0.52**	− 0.13	0.10	0.26	0.74
PERMA R1	− 0.14	**0.80**	0.02	0.53	0.47
PERMA R2	0.05	**0.80**	0.02	0.71	0.29
PERMA R3	0.15	**0.59**	0.09	0.54	0.46
PERMA P1	**0.36**	0.25	**0.36**	0.66	0.34
PERMA P2	**0.41**	0.11	**0.43**	0.66	0.34
PERMA E3	− 0.11	0.02	**0.48**	0.19	0.81

F1–F3: standardized factor loadings from the three-factor model extracted using the principal axis factoring method and oblique promax rotation. The loadings represent each item’s contribution to the corresponding factor; absolute values ≥ 0.30 are generally considered significant, ≥ 0.50 as strong. H^2^ (communalities): proportion of variance in each item explained by all factors in the model. High values (≥ 0.40) indicate that the item is well represented by the factor solution. U^2^ (uniqueness): proportion of variance not explained by the factors, including specific error or variance unique to the item. High uniqueness (≥ 0.60) may suggest a weakly saturated item or conceptually distinct content. Moderate to strong positive correlations indicate the interdependence of latent dimensions and support the relevance of a hierarchical confirmatory model (specific factors + general factor). Bold values indicate the highest factor loading(s) for each item. Items with similar loadings across multiple factors (e.g., P1, P2) suggest potential cross-loadings.

Finally, the residual matrix of the 3-factor model (online Appendix C1) shows generally small differences between the observed matrix and the reproduced matrix, supporting the overall adequacy of this exploratory solution. Based on these considerations, confirmatory analyses were restricted to theoretically and psychometrically interpretable models, including the 5-factor structure and its hierarchical extension. All detailed results relating to the CFA (including factor loadings, communalities, fit indices, inter-factor correlations, and parallel analysis results) are available in the supplementary material (Appendices C2–C5).

### Confirmatory factor analysis

Confirmatory factor analyses (CFA) were performed to validate the latent structure of the French version of the PERMA-Profiler, identified during the EFA. In accordance with current methodological recommendations in psychometrics, confirmatory analyses were conducted on the independent G2 subsample (n = 306), ensuring strict cross-validation and limiting the risk of model overfitting.

The CFA was performed on the 15 items of the PERMA-Profiler corresponding to the five theoretical dimensions of the model (Positive Emotion, Engagement, Relationships, Meaning, Accomplishment). The analyses were conducted on complete-case data for all 15 items, resulting in a final sample size of N = 306 participants in G2.

Given the ordinal format of the items (11-point Likert scales), the main estimation was performed using the WLSMV estimator, recommended for non-normally distributed ordinal variables. In order to assess the robustness of the results, a sensitivity analysis was also conducted by treating the items as continuous, using the MLR estimator (see online Appendix D5).

Four confirmatory models were specified and compared: (1) a one-factor model, positing a single general factor of well-being; (2) a 3-factor model, corresponding to the parsimonious structure suggested by the EFA; (3) a 5-factor model, corresponding to the theoretical structure of the PERMA Profiler; (4) a second-order hierarchical model, in which the five first-order factors load onto a general factor of well-being. The 3-factor model was included in the CFA framework to allow a direct comparison between a parsimonious data-driven solution and theoretically grounded models, in line with cross-validation principles in psychometrics. A bifactor model (i.e., a general factor and five orthogonal specific factors) was also explored on an exploratory basis. However, this model did not converge satisfactorily with the WLSMV estimator and presented identification problems and negative variances with the MLR estimator. As a result, it was not retained for interpretation.

The fit indices for the models tested with the WLSMV estimator are presented in Table 6. Standard interpretation thresholds were used (CFI/TLI ≥ 0.95; RMSEA ≤ 0.08; SRMR ≤ 0.08). The 1-factor model shows insufficient fit, with a high RMSEA value (RMSEA = 0.107) despite high incremental indices, suggesting an overly simplified construct structure. The 3-factor model showed inadequate fit according to conventional thresholds (CFI = 0.975; TLI = 0.970; RMSEA = 0.096; SRMR = 0.046), particularly due to the RMSEA value exceeding recommended limits, despite acceptable incremental indices. In addition, inspection of the standardized loadings confirmed the interpretability limitations already identified in the EFA. The third factor was weakly defined, with E3 showing a low loading, while other items (e.g., P1 and P2) displayed cross-loadings across multiple factors. This pattern indicates that the third factor does not represent a coherent or theoretically meaningful latent construct. The 5-factor correlated model shows an excellent fit to the data (CFI = 0.996; TLI = 0.995; RMSEA = 0.064), indicating that the multidimensional structure of PERMA is well supported empirically in the G2 sample. The second-order hierarchical model also shows a good overall fit, slightly lower than that of the five-factor correlated model, but consistent with the hypothesis of a general well-being factor structuring the five specific dimensions. Detailed results of the fit indices are presented in Table 6.

**Table 6:** Fit indices for the tested CFA models (G2, WLSMV estimator).

	1-factor	3-factors	5 correlated factors	Second order (5 → 1)
χ^2^ (df)	403.92 (90)	333.70 (87)	178.43 (80)	198.30 (85)
CFI	0.987	0.975	0.996	0.995
TLI	0.985	0.970	0.995	0.994
RMSEA [90% CI]	0.107 [0.096–0.118]	0.096 [0.086–0.107]	0.064 [0.051–0.076]	0.066 [0.054–0.078]
SRMR	0.067	0.046	0.041	0.045

*Χ*^2^, chi-square of the model; CFI, Comparative Fit Index; TLI, Tucker-Lewis Index; RMSEA, Root Mean Square Error of Approximation; SRMR, Standardized Root Mean Square Residual. The conventional adjustment thresholds used are CFI/TLI ≥ 0.90 and RMSEA/SRMR ≤ 0.08. All p values are < 0.001.

The standardized factor loadings of the five-factor correlated model are all substantial and statistically significant. The R^2^ values indicate a satisfactory proportion of variance explained by the latent factors for all items. The standardized factor loadings and coefficients of determination (R^2^) are presented in detail in online Appendix D1. The inter-factor correlations (Φ matrix) are reported in online Appendix D2, showing moderate to high associations between the dimensions, consistent with the hypothesis of a strongly interrelated multidimensional construct. The standardized residual matrices (online Appendix D3) do not reveal any systematic patterns of local misspecification.

The analyses performed with the MLR estimator, treating the items as continuous, lead to convergent results, with an overall fit that is slightly lower but qualitatively similar to that obtained with WLSMV (see online Appendix D4). This convergence supports the robustness of the conclusions regarding the factorial structure of the PERMA-Profiler.

Overall, the CFA results clearly support the 5-factor correlated model as the most adequate representation of the PERMA-Profiler structure in this sample. Although the 3-factor solution emerged in the exploratory analyses as a parsimonious alternative, its confirmatory evaluation confirmed that it does not meet standard criteria for a well-defined latent structure. Specifically, the third factor remained poorly defined and lacked conceptual coherence, as it combined items from different theoretical dimensions with substantial cross-loadings and uneven saturation. Importantly, a latent factor defined by a single salient indicator does not meet standard psychometric criteria for factorial validity, further supporting the rejection of the 3-factor solution. Taken together, these results support the conclusion that the 3-factor solution is not an adequate representation of the latent structure of the PERMA-Profiler in this sample, despite its relative parsimony. The second-order hierarchical model is a conceptually relevant alternative when the use of an overall well-being score is justified, but the 5-factor correlated model is retained as the main model for further analyses.

### Internal consistency

The internal consistency of the PERMA-Profiler was examined in the total sample (N = 612), as well as separately in the G1 (EFA) and G2 (CFA) subsamples (n = 306 each) using both Cronbach’s alpha (α) and McDonald’s omega (ω). In accordance with current methodological recommendations, omega, estimated from the chosen confirmatory model, was preferred as the main reliability index, with alpha reported as a supplement.

Reliability coefficients were calculated for each of the five dimensions of the PERMA model (Positive Emotion, Engagement, Relationships, Meaning, Accomplishment), as well as for the total score, across the total sample and both subsamples. Cronbach’s alpha and McDonald’s omega coefficients for each dimension and sample are presented in Table 7.

**Table 7:** Internal consistency of the PERMA-Profiler across total sample and subsamples.

Dimension	n items	Sample	N	α	ω
Positive emotion (P)	3	Total	612	0.870	0.870
		G1	306	0.837	0.838
		G2	306	0.896	0.897
Engagement (E)	3	Total	612	0.530	0.571
		G1	306	0.525	0.557
		G2	306	0.541	0.589
Relationships (R)	3	Total	612	0.784	0.794
		G1	306	0.766	0.776
		G2	306	0.801	0.814
Meaning (M)	3	Total	612	0.906	0.906
		G1	306	0.890	0.890
		G2	306	0.919	0.919
Accomplishment (A)	3	Total	612	0.678	0.690
		G1	306	0.636	0.677
		G2	306	0.711	0.728
Total score	15	Total	612	0.913	0.921
		G1	306	0.898	0.908
		G2	306	0.925	0.932

*α* = Cronbach’s alpha. ω = Mcdonald’s omega. Reliability estimates are reported for the total sample and separately for the exploratory (G1) and confirmatory (G2) subsamples.

Cronbach’s alpha coefficients indicated good to excellent internal consistency for most dimensions across samples, particularly for Positive emotion (α = 0.84–0.90), Relationships (α = 0.77–0.80), Meaning (α = 0.89–0.92) and the total score (α = 0.90–0.92). The Accomplishment dimension demonstrated lower internal consistency (α = 0.52–0.54). Mcdonald’s omega coefficients showed a similar pattern, supporting the robustness of these findings. Importantly, reliability estimates were highly consistent across the total sample and both subsamples, supporting the stability of the PERMA-Profiler structure across independent datasets.

Overall, the results indicate satisfactory to excellent internal consistency for most dimensions. The lower reliability observed for the Engagement dimension is consistent with previous PERMA-Profiler validation studies and may reflect greater conceptual heterogeneity of the items comprising this subscale, rather than a limitation of the overall model. This dimension is therefore interpreted with caution in subsequent analyses.

### Convergent and discriminant validity and associated regression analyses

The validity of the PERMA-Profiler was examined in the validation sample (G2, N = 306) through correlational analyses and regression models, in accordance with methodological recommendations for the validation of multidimensional psychometric instruments. Analyses were structured to distinguish between convergent and discriminant validity and exploratory regression models.

Convergent validity was assessed by examining associations between PERMA-Profiler scores and an external theoretically related measure of psychological well-being, namely flourishing (FS total score). Additional indicators including perceived happiness, physical health, loneliness, and negative emotions were also examined. These variables correspond to single-item components included within the PERMA-Profiler itself and were therefore treated as complementary internal indicators rather than independent external validation measures. As such, associations involving these variables are reported descriptively and are not interpreted as formal tests of convergent validity.

Pearson correlation analyses showed that all PERMA dimensions were positively and significantly associated with flourishing (see Table 8). The total PERMA-Profiler score showed a strong correlations with flourishing, supporting the existence of a general well-being factor. These results confirm the convergent validity of the PERMA-Profiler in the sample studied. Detailed correlation statistics (including confidence intervals and sample sizes) are reported in online Appendix E1.

**Table 8:** Correlations between PERMA-Profiler scores and validation variables (G2).

Variable	Total	P	E	R	M	A
Flourishing	0.84***	0.75***	0.51***	0.61***	0.82***	0.69***
HADS anxiety	− 0.44***	− 0.51***	− 0.17**	− 0.30***	− 0.38***	− 0.38***
HADS depression	− 0.67***	− 0.66***	− 0.39***	− 0.53***	− 0.56***	− 0.53***
Negative emotions	− 0.49***	− 0.59***	− 0.22***	− 0.31***	− 0.41***	− 0.42***
Loneliness	− 0.50***	− 0.49***	− 0.20***	− 0.49***	− 0.43***	− 0.35***
Perceived happiness	0.87***	0.86***	0.55***	0.67***	0.75***	0.55***
Health	0.49***	0.46***	0.28***	0.41***	0.42***	0.38***

**p < 0.01, ***p < 0.001. Values represent Pearson correlations. Sample sizes ranged from 292 to 306 depending on missing data. Flourishing and HADS anxiety and depression represent external validation measures. Perceived happiness, health, loneliness, and negative emotions correspond to additional single-item indicators included within the PERMA-Profiler and are reported as complementary descriptive indicators rather than independent external validation measures.

Discriminant validity was assessed through associations between PERMA-Profiler scores and theoretically distinct measures of psychological distress, namely HADS anxiety and depression scores. As expected, all correlations with HADS scores were negative and statistically significant (see Table 8). Associations with anxiety were generally small to moderate in magnitude, whereas several correlations with depressive symptoms reached moderate to strong magnitude, particularly for the overall PERMA score and the Positive Emotion dimension. However, these associations remained lower than those observed with flourishing, supporting the relative distinction between psychological well-being and psychological distress. Additional PERMA items, including negative emotions, loneliness and health, were examined separately as complementary internal indicators and were not interpreted as independent measures of discriminant validity.

Regression analyses were conducted to explore the extent to which PERMA scores were associated with variance in external well-being and distress indicators. Two sets of adjusted models were estimated. Table 9 presents models in which the overall PERMA score was entered as a predictor of external criteria (Flourishing, HADS Anxiety, HADS Depression, Negative emotion, Loneliness, Perceived Happiness and Health), controlling for age, gender and education. The models explained a substantial proportion of variance in the outcome variables, with adjusted R^2^ values ranging from 0.23 to 0.67. Table 10 presents models in which the five PERMA dimensions were entered simultaneously as predictors of these same external criteria, again controlling for age, gender and education. In multivariable models including all five PERMA dimensions simultaneously, the proportion of explained variance remained substantial across outcomes, with adjusted R^2^ values ranging from 0.23 to 0.69. The inclusion of all PERMA dimensions in the same model resulted in attenuation of some individual coefficients, reflecting shared variance among dimensions and potential suppression effects.

**Table 9:** Adjusted regressions models predicting external well-being and distress indicators from overall PERMA well-being (G2). (Model: Y ~ Overall PERMA well-being + age + gender + education).

Criterion (Y)	Predictor	b	SE	t	[95% CI]	*p*
Flourishing Scale (FS) adjusted R^2^ = 0.67	Overall PERMA well-being	5.227	0.182	28.70	[4.868, 5.585]	< 0.001
	Age	0.038	0.019	1.95	[0.000, 0.076]	0.052
	Gender: Woman	− 4.447	3.104	− 1.43	[− 10.558, 1.664]	0.153
	Gender: Man	− 4.906	3.120	− 1.57	[− 11.047, 1.235]	0.117
	Education: Secondary school	− 2.145	2.020	− 1.06	[− 6.122, 1.832]	0.289
	Education: High school	− 1.462	0.858	− 1.70	[− 3.150, 0.227]	0.089
	Education: University student	0.151	1.251	0.12	[− 2.310, 2.613]	0.904
	Education: Masters	− 0.278	0.715	− 0.39	[− 1.686, 1.130]	0.698
	Education: Doctorate	− 0.351	0.948	− 0.37	[− 2.217, 1.515]	0.712
Anxiety (HADS-A) adjusted R^2^ = 0.26	Overall PERMA well-being	− 1.358	0.138	− 9.84	[− 1.630, − 1.086]	< 0.001
	Age	− 0.044	0.015	− 3.02	[− 0.073, − 0.015]	0.003
	Gender: Woman	− 2.713	2.356	− 1.15	[− 7.350, 1.925]	0.251
	Gender: Man	− 3.730	2.368	− 1.58	[− 8.390, 0.931]	0.116
	Education: Secondary school	2.992	1.532	1.95	[− 0.025, 6.008]	0.052
	Education: High school	− 0.270	0.645	− 0.42	[− 1.539, 0.999]	0.676
	Education: University student	0.403	0.948	0.42	[− 1.462, 2.268]	0.671
	Education: Masters	− 1.077	0.538	− 2.00	[− 2.137, − 0.017]	0.046
	Education: Doctorate	− 1.663	0.712	− 2.34	[− 3.063, − 0.262]	0.020
Depression (HADS-D) adjusted R^2^ = 0.46	Overall PERMA well-being	− 1.647	0.096	− 17.09	[− 1.837, − 1.457]	< 0.001
	Age	0.001	0.010	0.05	[− 0.020, 0.021]	0.959
	Gender: Woman	1.371	1.645	0.83	[− 1.866, 4.609]	0.405
	Gender: Man	1.029	1.653	0.62	[− 2.224, 4.283]	0.534
	Education: Secondary school	− 0.229	1.070	− 0.21	[− 2.334, 1.877]	0.831
	Education: High school	0.068	0.450	0.15	[− 0.818, 0.954]	0.881
	Education: University student	− 0.056	0.661	− 0.08	[− 1.358, 1.246]	0.933
	Education: Masters	− 0.696	0.376	− 1.85	[− 1.436, 0.044]	0.065
	Education: Doctorate	− 0.077	0.497	− 0.15	[− 1.054, 0.901]	0.878
Negative emotion adjusted R^2^ = 0.34	Overall PERMA well-being	− 0.664	0.064	− 10.44	[− 0.790, − 0.539]	< 0.001
	Age	− 0.007	0.007	− 0.99	[− 0.020, 0.007]	0.321
	Gender: Woman	0.752	1.105	0.68	[− 1.422, 2.926]	0.496
	Gender: Man	0.440	1.110	0.40	[− 1.745, 2.624]	0.692
	Education: Secondary school	1.315	0.717	1.83	[− 0.097, 2.726]	0.068
	Education: High school	− 0.130	0.295	− 0.44	[− 0.710, 0.450]	0.659
	Education: University student	0.784	0.442	1.77	[− .086, 1.655]	0.077
	Education: Masters	− 0.107	0.249	− 0.43	[− 0.597, 0.392]	0.666
	Education: Doctorate	− 0.374	0.329	− 1.14	[− 1.020, 0.273]	0.256
Loneliness adjusted R^2^ = 0.23	Overall PERMA well-being	− 1.058	0.106	− 10.01	[− 1.266, − 0.850]	< 0.001
	Age	0.013	0.011	1.14	[− 0.009, 0.035]	0.257
	Gender: Woman	− 1.500	1.834	− 0.82	[− 5.110, 2.110]	0.414
	Gender: Man	− 1.053	1.843	− 0.57	[− 4.680, 2.574]	0.568
	Education: Secondary school	0.550	1.191	0.46	[− 1.794, 2.894]	0.645
	Education: High school	0.997	0.489	2.04	[0.034, 1.960]	0.042
	Education: University student	0.731	0.735	0.99	[− 0.715, 2.177]	0.321
	Education: Masters	0.263	0.413	0.64	[− 0.549, 1.076]	0.524
	Education: Doctorate	− 0.256	0.545	− 0.47	[− 1.330, 0.817]	0.639
Perceived happiness adjusted R^2^ = 0.67	Overall PERMA well-being	1.303	0.044	29.65	[1.216, 1.389]	< 0.001
	Age	0.002	0.005	0.42	[− 0.007, 0.011]	0.672
	Gender: Woman	− 0.413	0.763	− 0.54	[− 1.914, 1.089]	0.589
	Gender: Man	− 0.601	0.767	− 0.78	[− 2.110, 0.908]	0.434
	Education: Secondary school	0.010	0.495	0.02	[− 0.065, 0.985]	0.984
	Education: High school	− 0.047	0.204	− 0.23	[− 0.447, 0.354]	0.819
	Education: University student	0.353	0.306	1.15	[− 0.249, 0.954]	0.249
	Education: Masters	− 0.112	0.172	− 0.65	[− 0.450, 0.226]	0.516
	Education: Doctorate	− 0.011	0.227	− 0.05	[− 0.458, 0.435]	0.961
Health adjusted R^2^ = 0.23	Overall PERMA well-being	0.560	0.075	7.52	[0.413, 0.707]	< 0.001
	Age	0.013	0.008	1.66	[− 0.002, 0.029]	0.098
	Gender: Woman	0.319	1.294	0.25	[− 2.229, 2.866]	0.806
	Gender: Man	0.610	1.301	0.47	[− 1.950, 3.169]	0.639
	Education: Secondary school	− 0.435	0.840	− 0.52	[− 2.089, 1.219]	0.605
	Education: High school	0.109	0.345	0.32	[− 0.571, 0.788]	0.753
	Education: University student	0.095	0.518	0.18	[− 0.926, 1.115]	0.855
	Education: Masters	0.282	0.291	0.97	[− 0.291, 0.856]	0.334
	Education: Doctorate	− 0.039	0.385	− 0.10	[− 0.796, 0.719]	0.920

Adjusted linear regression models were estimated in the validation sample (G2) to examine associations between overall PERMA well-being and external well-being and distress indicators, controlling for age, gender and education. b = unstandardized regression coefficient; SE = standard error; 95% CI = 95% confidence interval. Adjusted R^2^ represents the proportion of variance explained by the model, adjusted for the number of predictors. For gender, “Other” was the reference category. For education, “Bachelors” was the reference category. The category Primary school was not represented in the G2 regression sample and was therefore not estimated.

**Table 10:** Adjusted regression models predicting external well-being and distress indicators from the five PERMA dimensions entered simultaneously (G2). (Model: Y ~ P + E + R + M + A + age + gender + education).

Criterion (Y)	Predictor	b	SE	t	95% CI	*p*
Flourishing Scale (FS) adjusted R^2^ = 0.69	Positive emotion (P)	1.597	0.253	6.30	[1.098, 2.096]	< 0.001
	Engagement (E)	− 0.058	0.231	− 0.25	[− 0.513, 0.397]	0.801
	Relationships (R)	0.787	0.188	4.20	[0.418, 1.156]	< 0.001
	Meaning (M)	1.595	0.226	7.07	[1.151, 2.039]	< 0.001
	Accomplishment (A)	0.916	0.250	3.66	[0.424, 1.409]	< 0.001
	Age	0.026	0.019	1.34	[− 0.012, 0.064]	0.180
	Gender: Woman	− 4.856	3.049	− 1.59	[− 10.857, 1.146]	0.112
	Gender: Man	− 5.144	3.057	− 1.68	[− 11.162, 0.873]	0.094
	Education: Secondary school	− 0.447	1.988	− 0.22	[− 4.360, 3.466]	0.822
	Education: High school	− 0.733	0.857	− 0.86	[− 2.420, 0.954]	0.393
	Education: University student	1.018	1.224	0.83	[− 1.391, 3.426]	0.406
	Education: Masters	− 0.106	0.695	− 0.15	[− 1.475, 1.263]	0.879
	Education: Doctorate	− 0.557	0.926	− 0.60	[− 2.380, 1.267]	0.548
Anxiety (HADS-A) adjusted R^2^ = 0.33	Positive Emotion (P)	− 1.254	0.188	− 6.68	[− 1.624, − 0.885]	< 0.001
	Engagement ®	0.277	0.172	1.61	[− 0.061, 0.615]	0.108
	Relationships ®	− 0.117	0.138	− 0.85	[− 0.389, 0.155]	0.398
	Meaning (M)	0.218	0.167	1.30	[− 0.111, 0.547]	0.194
	Accomplishment (A)	− 0.420	0.185	− 2.27	[− 0.783, − 0.056]	0.024
	Age	− 0.041	0.014	− 2.87	[− 0.069, − 0.013]	0.004
	Gender : Woman	− 2.040	2.272	− 0.90	[− 6.512, 2.432]	0.370
	Gender : Man	− 3.088	2.279	− 1.36	[− 7.574, 1.397]	0.176
	Education : Secondary school	1.912	1.482	1.29	[− 1.005, 4.828]	0.198
	Education : High school	− 0.096	0.634	− 0.15	[− 1.344, 1.152]	0.880
	Education : University student	0.300	0.911	0.33	[− 1.494, 2.093]	0.742
	Education : Masters	− 1.149	0.514	− 2.23	[− 2.162, − 0.137]	0.026
	Education : Doctorate	− 1.686	0.683	− 2.47	[− 3.031, − 0.340]	0.014
Depression (HADS-D) adjusted R^2^ = 0.46	Positive Emotion (P)	− 0.989	0.133	− 7.42	[− 1.251, − 0.727]	< 0.001
	Engagement (E)	0.062	0.122	0.51	[− 0.178, 0.302]	0.612
	Relationships (R)	− 0.242	0.098	− 2.47	[− 0.436, − 0.049]	0.014
	Meaning (M)	− 0.128	0.119	− 1.08	[− 0.362, 0.106]	0.282
	Accomplishment (A)	− 0.252	0.131	− 1.92	[− 0.510, 0.006]	0.056
	Age	0.002	0.010	0.24	[− 0.017, 0.022]	0.810
	Gender: Woman	1.634	1.613	1.01	[− 1.541, 4.809]	0.312
	Gender: Man	1.281	1.618	0.79	[− 1.903, 4.465]	0.429
	Education: Secondary school	− 0.978	1.052	− 0.93	[− 3.048, 1.092]	0.353
	Education: High school	0.084	0.450	0.19	[− 0.802, 0.970]	0.852
	Education: University student	− 0.239	0.647	− 0.37	[− 1.512, 1.034]	0.712
	Education: Masters	− 0.739	0.365	− 2.02	[− 1.458, − 0.020]	0.044
	Education: Doctorate	− 0.120	0.485	− 0.25	[− 1.075, 0.835]	0.804
Negative emotion adjusted R^2^ = 0.40	Positive Emotion (P)	− 0.730	0.082	− 8.87	[− 0.892, − 0.568]	< 0.001
	Engagement (E)	0.193	0.076	2.55	[.044, 0.342]	0.011
	Relationships (R)	− 0.067	0.061	− 1.11	[− 0.187, 0.053]	0.270
	Meaning (M)	0.155	0.074	2.10	[0.010, 0.301]	0.037
	Accomplishment (A)	− 0.172	0.081	− 2.13	[− 0.331, − 0.013]	0.034
	Age	− 0.005	0.006	− 0.83	[− 0.018, 0.007]	0.407
	Gender: Woman	1.135	1.023	1.11	[− 0.879, 3.149]	0.268
	Gender: Man	0.821	1.026	0.80	[− 1.199, 2.841]	0.425
	Education: Secondary school	0.736	0.666	1.11	[− 0.574, 2.046]	0.270
	Education: High school	− 0.010	0.278	− 0.04	[− 0.557, 0.537]	0.972
	Education: University student	0.745	0.409	1.82	[− 0.060, 1.549]	0.070
	Education: Masters	− 0.105	0.228	− 0.46	[− 0.555, 0.344]	0.646
	Education: Doctorate	− 0.352	0.303	− 1.16	[− 0.948, 0.245]	0.247
Loneliness adjusted R^2^ = 0.27	Positive Emotion (P)	− 0.451	0.138	− 3.26	[− 0.723, − 0.179]	0.001
	Engagement (E)	0.075	0.127	0.59	[− 0.175, 0.325]	0.556
	Relationships (R)	− 0.855	0.102	− 8.35	[− 1.056, − 0.653]	< 0.001
	Meaning (M)	0.342	0.125	2.74	[0.096, 0.587]	0.006
	Accomplishment (A)	− 0.253	0.136	− 1.87	[− 0.520, 0.014]	0.063
	Age	0.002	0.011	0.18	[− 0.019, 0.023]	0.860
	Gender: Woman	− 0.147	1.718	− 0.09	[− 3.529, 3.235]	0.932
	Gender: Man	0.264	1.723	0.15	[− 3.128, 3.656]	0.878
	Education: Secondary school	0.824	1.118	0.74	[− 1.376, 3.024]	0.462
	Education: High school	1.081	0.467	2.32	[0.163, 2.000]	0.021
	Education: University student	0.856	0.686	1.25	[− 0.495, 2.207]	0.213
	Education: Masters	0.363	0.384	0.95	[− 0.392, 1.118]	0.345
	Education: Doctorate	− 0.054	0.509	− 0.11	[− 1.056, 0.948]	0.916
Perceived happiness adjusted R^2^ = 0.68	Positive Emotion (P)	0.777	0.058	13.32	[0.662, 0.892]	< .001
	Engagement ®	0.001	0.054	0.03	[− 0.104, 0.107]	0.979
	Relationships ®	0.244	0.043	5.65	[0.159, 0.329]	< 0.001
	Meaning (M)	0.177	0.053	3.37	[0.074, 0.281]	< 0.001
	Accomplishment (A)	− 0.049	0.057	− 0.86	[− 0.162, 0.063]	0.388
	Age	0.003	0.005	0.61	[− 0.006, 0.012]	0.541
	Gender : Woman	− 0.296	0.725	− 0.41	[− 1.724, 1.131]	0.683
	Gender : Man	− 0.505	0.727	− 0.69	[− 1.937, 0.927]	0.488
	Education : Secondary school	0.466	0.472	0.99	[− 0.462, 1.395]	0.324
	Education : High school	− 0.040	0.197	− 0.20	[− 0.427, 0.348]	0.840
	Education : University student	0.496	0.290	1.71	[− 0.074, 1.066]	0.088
	Education : Masters	− 0.138	0.162	− 0.85	[− 0.457, 0.180]	0.393
	Education : Doctorate	0.051	0.215	0.24	[− 0.372, 0.473]	0.814
Health adjusted R^2^ = 0.23	Positive Emotion (P)	0.555	0.102	5.43	[0.353, 0.756]	< 0.001
	Engagement (E)	− 0.064	0.094	− 0.68	[− 0.249, 0.121]	0.495
	Relationships (R)	− 0.059	0.076	− 0.78	[− 0.208, 0.090]	0.434
	Meaning (M)	− 0.074	0.092	− 0.80	[− 0.255, 0.107]	0.422
	Accomplishment (A)	0.184	0.100	1.84	[− 0.013, 0.382]	0.067
	Age	0.010	0.008	1.31	[− 0.005, 0.026]	0.192
	Gender: Woman	0.218	1.271	0.17	[− 2.283, 2.719]	0.864
	Gender: Man	0.492	1.274	0.39	[− 2.016, 3.000]	0.700
	Education: Secondary school	0.042	0.827	0.05	[− 1.584, 1.669]	0.959
	Education: High school	− 0.029	0.345	− 0.08	[− 0.708, 0.650]	0.933
	Education: University student	0.108	0.508	0.21	[− 0.891, 1.107]	0.832
	Education: Masters	0.289	0.284	1.02	[− 0.269, 0.848]	0.308
	Education: Doctorate	− 0.020	0.376	− 0.05	[− 0.761, 0.721]	0.958

Adjusted linear regression models were estimated in the validation sample (G2) to examine associations between the five PERMA dimensions entered simultaneously and external well-being and distress indicators, controlling for age, gender and education. b, unstandardized regression coefficient; SE, standard error; 95% CI, 95% confidence interval. Adjusted R^2^ represents the proportion of variance explained by the model, adjusted for the number of predictors. For gender, « Other** »** was the reference category. For education, « Bachelors** »** was the reference category. The category « Primary school** »** was not represented in the G2 regression sample and was therefore not estimated.

These analyses were not intended to assess criterion validity but should be interpreted as exploratory models examining predictive associations between PERMA scores and external variables. The results indicate that the overall PERMA score was significantly associated with all external criteria and that several PERMA dimensions retained significant associations in the multivariable models, highlighting their incremental contribution within a multidimensional framework.

Importantly, the dependent variables consisted of either external measures (e.g., flourishing, HADS) or non-core PERMA items that were not part of the PERMA total score, thereby limiting direct mathematical overlap between predictors and outcomes.

### Test–retest reliability and temporal stability

The temporal stability of the PERMA-Profiler was examined in the matched sample using data collected at two measurement points (T1 and T2). Responses were individually matched using a unique identifier. The main analyses were conducted on the full matched sample, including all participants with available data at both time points (N ranging from 175 to 179 depending on the dimension).

Temporal stability was assessed at the scale level based on the mean scores of the PERMA model subdimensions (Positive Emotions, Engagement, Relationships, Meaning, Accomplishment), as well as the total score. For each score, test–retest reliability was estimated using the intraclass correlation coefficient ICC(2,1), corresponding to a two-way random effects model with absolute agreement and single measurement. In addition, Pearson’s correlation (r) between T1 and T2 was calculated to facilitate comparison with the existing literature. 95% confidence intervals were estimated for all coefficients. Absolute error indices were also calculated, including the standard error of measurement (SEM) and the smallest detectable change at 95% (SDC95), in order to document the accuracy of the longitudinal scores.

The results for the scales are presented in Table 11. Overall, temporal stability appears to be good to excellent for most of the PERMA-Profiler dimensions. The Positive Emotions, Relationships and Meaning subscales, as well as the total score, have high ICC(2,1) coefficients (≥ 0.83), indicating high stability over the time interval considered. The Achievement dimension also shows satisfactory stability. In contrast, the Engagement dimension shows more moderate stability, with an ICC coefficient lower than the other subscales, suggesting greater intra-individual variability over time. Pearson’s correlation coefficients follow a similar pattern and confirm the consistency of the results obtained with the ICC.

**Table 11:** Temporal stability of the PERMA Profiler (matched sample).

Dimension	ICC(2,1)	[95% CI]	r	[95% CI]	SEM	SDC95
Positive emotion (P)	0.844	[0.80; 0.88]	0.843	[0.79; 0.88]	0.650	1.80
Engagement (E)	0.691	[0.60; 0.76]	0.695	[0.61; 0.76]	0.782	2.17
Relationships (R)	0.776	[0.71; 0.83]	0.776	[0.71; 0.83]	0.755	2.09
Meaning (M)	0.813	[0.76; 0.86]	0.814	[0.76; 0.86]	0.799	2.22
Accomplishment (A)	0.780	[0.71; 0.83]	0.780	[0.72; 0.83]	0.731	1.98
Score total	0.856	[0.81; 0.89]	0.855	[0.81; 0.89]	0.494	1.37

ICC(2,1), intraclass correlation coefficient (bidirectional random effects model, absolute agreement, single measurement). r, Pearson correlation between T1 and T2. SEM, standard error of measurement. SDC95, smallest detectable change at 95%. Values are based on the full matched sample.

To further account for the cross-validation structure of the study, test–retest reliability of the PERMA total score was also examined separately in participants originating from the G1 and G2 subsamples. The correlation between T1 and T2 was strong in the full matched sample (r = 0.86, 95% CI [0.82, 0.90], *p* < 0.001) and remained substantial in G1 (r = 0.78) and G2 (r = 0.92), supporting the robustness of temporal stability across subsamples (see online Appendix F1).

The SEM and SDC95 values indicate that, for most dimensions, the changes observed must reach a moderate amplitude to be interpreted as exceeding the measurement error. The thresholds for detectable change are higher for the Engagement dimension, which is consistent with its relatively lower stability and suggests increased sensitivity to contextual or situational fluctuations.

Overall, these results indicate that the PERMA-Profiler exhibits satisfactory temporal stability at the scale level, particularly for the Positive Emotions, Relationships, and Meaning dimensions, as well as for the total score. The more moderate stability observed for the Engagement dimension is consistent with the results of factor and internal consistency analyses, and suggests that this dimension may reflect components that are more sensitive to contextual variations or transient states.

## Discussion

The aims of this study was to translate and culturally adapt the PERMA-Profiler scale into French, then rigorously evaluate its psychometric properties in a large adult sample. The results provide strong support for the validity of the French version of the instrument and are consistent with the international literature on multidimensional measurement of psychological well-being within the PERMA model.

Methodologically, this study stands out for the rigor of its validation process. The linguistic adaptation process was based on an explicit comparison of alternative formulations for certain items, combining theoretical expertise and back-translation procedures to ensure high conceptual fidelity to the original version^24^. Furthermore, the strict separation between the exploratory and confirmatory phases, based on two independent and statistically comparable subsamples, meets contemporary standards in psychometrics and limits the risk of model overfitting. This strategy lends particular robustness to the factorial conclusions.

Exploratory factor analyses reveal a latent structure characterized by strong interdependence among the components of well-being. Parallel analysis suggests a three-factor solution, and comparison of exploratory models shows that a one-dimensional structure is clearly insufficient. However, the five-factor theoretical model presents the best overall indices and the highest explained variance at the exploratory stage. This type of configuration has already been reported in several international validations of the PERMA-Profiler, where more parsimonious exploratory solutions coexist with a better confirmatory fit of the theoretical model^26,67,68^. These results suggest that, although the dimensions of well-being share substantial variance, multidimensional modeling remains conceptually and empirically more faithful to the PERMA framework when tested confirmatorily.

Confirmatory factor analyses provide a central and robust result: the five-factor correlated model, corresponding to the dimensions of Positive Emotions, Engagement, Relationships, Meaning, and Accomplishment, fits the data very well and clearly outperforms the unifactorial model. The second-order hierarchical model, which posits the existence of a general well-being factor structuring the five specific dimensions, also shows a good fit, albeit slightly inferior. This configuration is fully consistent with the initial theoretical conception of PERMA, according to which well-being is conceived as a multidimensional construct articulated around distinct but interdependent pillars^23^. It also supports the conclusions of the original validation and several cross-cultural studies supporting the complementary use of specific sub-scores and an overall score, depending on the analytical objectives^24,69^.

The internal consistency analysis broadly confirms the psychometric soundness of the instrument. The PERMA-Profiler total score shows excellent reliability, and several subscales, particularly Positive Emotions, Relationships, and Meaning, show high indices comparable to those reported in other validations. The Accomplishment dimension shows acceptable reliability. In contrast, the Engagement dimension consistently stands out with lower internal consistency coefficients. This result is neither specific to the French version nor unexpected: more modest reliability for Engagement has been reported repeatedly in various studies, regardless of language or cultural context^26,67,68^. The convergence of these results invites critical reflection on the conceptual and psychometric status of Engagement in the PERMA model. Unlike the other dimensions, Engagement is closely linked to the experience of flow, defined as a state of optimal absorption, intrinsically dependent on context, activity, and moment^70^. Such experiences are, by nature, fluctuating and difficult to assess retrospectively. Several authors have pointed out that measuring engagement through general self-questionnaires tends to amalgamate heterogeneous components (i.e., behavioral involvement, subjective interest, cognitive absorption), which can undermine the internal consistency and temporal stability of this dimension^26^. Taken together, these results suggest that, within the PERMA model, Engagement does not function as a purely “trait-like” dimension of well-being, but rather as a more state-dependent component, whose measurement is particularly sensitive to contextual and situational variations.

The temporal stability results observed in this study reinforce this interpretation. While the total PERMA-Profiler score and the Positive Emotions, Relationships, and Meaning dimensions show good to excellent stability, Engagement shows more moderate stability. This profile suggests that Engagement reflects a facet of well-being that is more sensitive to intra-individual fluctuations related to opportunities for involvement and contextual constraints, rather than a relatively stable trait. Similar results have been reported in longitudinal studies on well-being, indicating that certain experiential or motivational dimensions show greater variability than more evaluative or existential dimensions^34,69^.

Convergent and discriminant validity analyses, together with regression analyses examining associations with external criteria, provide support for the conceptual validity of the PERMA-Profiler. As expected, PERMA dimensions and the overall score showed strong associations with flourishing and subjective perceived happiness, supporting convergent validity. Associations with anxiety and depression were negative and generally lower than those observed with flourishing, supporting discriminant validity. However, several correlations, particularly those involving depressive symptoms, reached moderate to strong magnitude. This finding indicates that the PERMA dimensions capture aspects of positive psychological functioning that are conceptually distinct from psychopathology, while also sharing substantial variance with depressive symptomatology. This pattern is consistent with dual continuum models of mental health, according to which well-being and mental illness are related but non-identical constructs^36^. The high predictive power of the PERMA total score after adjusting for sociodemographic variables reinforces its value as a summary indicator of well-being. However, when all five dimensions were entered simultaneously into regression models, the contribution of certain dimensions, particularly Engagement, appears attenuated despite theoretical consistent bivariate associations. This phenomenon is likely attributable to shared variance among the highly correlated PERMA dimensions and should therefore be interpreted with caution.

Several limitations of this study should be acknowledged. First, test–retest reliability analyses were performed on a moderate-sized subsample, which may limit the accuracy and generalizability of estimates of temporal stability. Second, the exclusive use of self-reported measures may introduce common variance related to the method, and future research would benefit from incorporating additional ecological, behavioral, or performance-based indicators, particularly for the engagement dimension, which appears to be particularly sensitive to contextual and situational factors. Furthermore, although convergent and discriminant validity were examined using theoretically related and more distant concepts, the study did not include an external measure specifically designed to assess discriminant validity using conceptually unrelated domains. While the relative magnitude of correlations provides useful information, future studies should incorporate more theoretically distant concepts (e.g., personality traits, value orientations, or cognitive styles) to more rigorously test the discriminant validity of the PERMA-Profiler. Finally, the invariance of the measure across sociodemographic or clinical subgroups has not been examined and represents an essential next step in extending the use of the PERMA-Profiler to intergroup comparisons.

In conclusion, this study provides strong evidence for the validity and reliability of the French version of the PERMA-Profiler. The results support the theoretical structure of five correlated factors, as well as the relevance of a general well-being factor. The Engagement dimension appears to be the most psychometrically fragile component, a finding that is widely shared in the literature and theoretically consistent with the experiential and contextual nature of this construct. Far from calling into question the PERMA framework, these results contribute to refining its operationalization and suggest that not all components of well-being are equivalent in terms of stability and trait-likeness. Finally, the variations observed according to certain sociodemographic characteristics, such as age or gender, are consistent with previous results and reinforce the external validity of the instrument in the general adult population.

## Supplementary Information

Below is the link to the electronic supplementary material.

Supplementary Material 1.

## Data Availability

The authors confirm that all experiments were carried out in compliance with current guidelines and regulations. French PERMA-Profiler scale is available free of charge at Open Science Framework and can be used by clinicians and scientists alike. All data has been made publicly available at 53t8c and can be accessed at [https://osf.io/53t8c].
