# Supplementary material for: PERMA-Profiler as a multidimensional measure of well-being in a French context
Source: Sci Rep. 2026 Jun 12;16:26861. doi: 10.1038/s41598-026-57354-z (PMC13518861; doi:10.1038/s41598-026-57354-z)
Supplement: Supplementary file 1 [file 41598_2026_57354_MOESM1_ESM.docx]

**Appendix A.** The final French version of the PERMA Profiler

List of PERMA-Profiler items in the original and the French version

| **Item No.** | **Label** | **Original version** | **French version** | **Response Anchors** |
| --- | --- | --- | --- | --- |
| 1 | A1* | How much of the time do you feel you are making progress toward accomplishing your goals? | Dans quelle mesure avez-vous l’impression que vous progressez vers l’accomplissement de vos objectifs ? | 0 = never (jamais), 10 = always (toujours) |
| 2 | P1 | In general, how often do you feel joyful? | En général, à quelle fréquence vous sentez-vous joyeux ? |  |
| 3 | N1 | In general, how often do you feel anxious? | En général, à quelle fréquence vous sentez-vous anxieux ? |  |
| 4 | A2 | How often do you achieve the important goals you have set for yourself? | A quelle fréquence atteignez-vous les objectifs importants que vous vous êtes fixés ? |  |
| 5 | H1 | In general, how would you say your health is? | En général, comment évalueriez-vous votre santé ? | 0 = terrible, 10 = excellent (excellente) |
| 6 | M1 | In general, to what extent do you lead a purposeful and meaningful life? | En général, dans quelle mesure menez-vous une vie utile et pleine de sens ? | 0 = not at all (pas du tout), 10 = completely (complètement) |
| 7 | R1 | To what extent do you receive help and support from others when you need it? | Dans quelle mesure recevez-vous de l’aide et du soutien de la part des autres lorsque vous en avez besoin ? |  |
| 8 | M2* | In general, to what extent do you feel that what you do in your life is valuable and worthwhile? | En général, dans quelle mesure pensez-vous que ce que vous faites dans votre vie a de la valeur et est digne d’intérêt ? |  |
| 9 | E2 | In general, to what extent do you feel excited and interested in things? | En général, dans quelle mesure vous sentez-vous excite et intéressé par les choses ? |  |
| 10 | Lon | How lonely do you feel in your daily life? | A quel point vous sentez-vous seul dans votre vie quotidienne ? |  |
| 11 | H2 | How satisfied are you with your current physical health? | Êtes-vous satisfait de votre santé physique actuelle ? |  |
| 12 | P2* | In general, how often do you feel positive? | En général, à quelle fréquence vous sentez-vous positif ? | 0 = never (jamais), 10 = always (toujours) |
| 13 | N2 | In general, how often do you feel angry? | En général, à quelle fréquence vous sentez-vous en colère ? |  |
| 14 | A3 | How often are you able to handle your responsibilities? | A quelle fréquence êtes-vous capable d’assumer vos responsabilités ? |  |
| 15 | N3 | In general, how often do you feel sad? | En général, à quelle fréquence vous sentez-vous triste ? |  |
| 16 | E3 | How often do you lose track of time while doing something you enjoy? | A quelle fréquence perdez-vous la notion du temps en faisant quelque chose que vous aimez ? |  |
| 17 | H3 | Compared to others of your same age and sex, how is your health? | En comparaison avec d’autres personnes du même âge et du même sexe, comment est votre santé ? | 0 = terrible, 10 = excellent (excellente) |
| 18 | R2 | To what extent do you feel loved? | Dans quelle mesure vous sentez-vous aimé ? | 0 = not at all (pas du tout), 10 = completely (complètement) |
| 19 | M3 | To what extent do you generally feel you have a sense of direction in your life? | Dans quelle mesure avez-vous généralement l’impression d’avoir un sens dans votre vie ? |  |
| 20 | R3 | How satisfied are you with your personal relationships? | Êtes-vous satisfait de vos relations personnelles ? |  |
| 21 | P3 | In general, to what extent do you feel contented? | En général, dans quelle mesure vous sentez-vous satisfait ? |  |
| 22 | hap | Taking all things together, how happy would you say you are? | En prenant tout en considération, diriez-vous que vous êtes heureux ? |  |

*Note. P: Positive emotions subscale; E: Engagement subscale; R: Relationships subscale; M: Meaning subscale; A: Accomplishment subscale; N: Negative emotions subscale; H: Health subscale; Lon: Loneliness single item; Hap: Happiness simple item. Items marked an asterisk were identified as posing difficulties in terms of wording during the translation process. Two alternative versions were submitted for back-translation, then reviewed by the committee of experts to ensure conceptual fidelity to the original version.*

**Appendix B.**

***Table B1.*** Descriptive statistics for PERMA-Profiler items (N = 612)

| **Item** | **Mean** | **SD** | **Min** | **Max** | **Skew.** | **Kurt.** |
| --- | --- | --- | --- | --- | --- | --- |
| P1 | 6.32 | 1.85 | 1.00 | 10.00 | -0.58 | 0.03 |
| P2 | 6.58 | 1.92 | 0.00 | 10.00 | -0.59 | -0.04 |
| P3 | 6.52 | 1.83 | 0.00 | 10.00 | -0.88 | 1.10 |
| E1 | 6.97 | 1.76 | 1.00 | 10.00 | -0.64 | 0.50 |
| E2 | 7.20 | 1.81 | 0.00 | 10.00 | -0.87 | 1.09 |
| E3 | 6.67 | 2.46 | 0.00 | 10.00 | -0.7 | -0.03 |
| R1 | 6.75 | 2.29 | 0.00 | 10.00 | -0.65 | -0.04 |
| R2 | 7.45 | 1.92 | 0.00 | 10.00 | -0.83 | 0.76 |
| R3 | 6.95 | 2.11 | 0.00 | 10.00 | -0.78 | 0.58 |
| M1 | 6.60 | 2.12 | 0.00 | 10.00 | -0.68 | 0.24 |
| M2 | 6.87 | 2.11 | 0.00 | 10.00 | -0.79 | 0.49 |
| M3 | 6.77 | 2.15 | 0.00 | 10.00 | -0.85 | 0.64 |
| A1 | 6.37 | 1.80 | 0.00 | 10.00 | -0.68 | 0.70 |
| A2 | 6.75 | 1.85 | 0.00 | 10.00 | -0.76 | 0.66 |
| A3 | 8.05 | 1.81 | 0.00 | 10.00 | -1.30 | 2.45 |
| N1 | 5.32 | 2.49 | 0.00 | 10.00 | -0.05 | -0.87 |
| N2 | 4.21 | 2.14 | 0.00 | 10.00 | 0.34 | -0.64 |
| N3 | 4.38 | 2.28 | 0.00 | 10.00 | 0.19 | -0.73 |
| H1 | 7.08 | 1.85 | 0.00 | 10.00 | -0.90 | 1.04 |
| H2 | 6.03 | 2.45 | 0.00 | 10.00 | -0.58 | -0.18 |
| H3 | 6.90 | 2.10 | 0.00 | 10.00 | -0.74 | 0.55 |
| Lon | 3.52 | 2.94 | 0.00 | 10.00 | 0.45 | -0.93 |
| Hap | 6.94 | 2.13 | 0.00 | 10.00 | -0.92 | 0.92 |

***Note.*** *All PERMA items are measured on a scale of 0–10. SD = standard deviation; Min = minimum; Max = maximum; Skew = skewness; Kurt = kurtosis.*

***Figure B1.*** Figure B3.1. Q–Q plot of standardized residuals (PERMA Total)

***Figure B2.*** Residual diagnostics: residuals vs. adjusted values, scale-location, leverage, and Cook's distances

***Figure B3.*** Additional residual diagnostics (model 3)

**Appendix C.**

***Table C1.*** Residual matrix (lower triangular) – 3-factor EFA model (G1)

|  | **P1** | **P2** | **P3** | **E1** | **E2** | **E3** | **R1** | **R2** | **R3** | **M1** | **M2** | **M3** | **A1** | **A2** | **A3** |
| --- | --- | --- | --- | --- | --- | --- | --- | --- | --- | --- | --- | --- | --- | --- | --- |
| **P1** | - |  |  |  |  |  |  |  |  |  |  |  |  |  |  |
| **P2** | 0.1136 | - |  |  |  |  |  |  |  |  |  |  |  |  |  |
| **P3** | 0.0214 | 0.0278 | - |  |  |  |  |  |  |  |  |  |  |  |  |
| **E1** | -0.0633 | -0.0169 | -0.0695 | - |  |  |  |  |  |  |  |  |  |  |  |
| **E2** | -0.0075 | -0.0201 | -0.0183 | 0.0651 | - |  |  |  |  |  |  |  |  |  |  |
| **E3** | -0.0393 | -0.0235 | -0.0395 | 0.0707 | 0.0738 | - |  |  |  |  |  |  |  |  |  |
| **R1** | 0.0048 | -0.0180 | -0.0560 | 0.0204 | 0.0067 | 0.0386 | - |  |  |  |  |  |  |  |  |
| **R2** | -0.0245 | -0.0089 | -0.0292 | 0.0482 | -0.0348 | -0.0075 | -0.0560 | - |  |  |  |  |  |  |  |
| **R3** | -0.0261 | -0.0492 | 0.1082 | -0.0476 | 0.0282 | -0.0109 | -0.0118 | 0.0262 | - |  |  |  |  |  |  |
| **M1** | 0.0229 | 0.0264 | -0.0200 | 0.0291 | -0.0269 | 0.0032 | -0.0281 | -0.0059 | -0.0281 | - |  |  |  |  |  |
| **M2** | 0.0113 | 0.0100 | -0.0128 | -0.0072 | 0.0656 | -0.0074 | -0.0257 | -0.0244 | -0.00598 | 0.00129 | - |  |  |  |  |
| **M3** | -0.0063 | 0.0109 | -0.0037 | 0.01031 | 0.01061 | 0.00528 | -0.0222 | -0.00598 | -0.0222 | -0.0281 | -0.00598 | - |  |  |  |
| **A1** | -0.0329 | -0.0444 | 0.0325 | 0.00075 | -0.00339 | -0.02833 | 0.0883 | 0.0362 | -0.0124 | 0.00129 | -0.02436 | -0.02575 | - |  |  |
| **A2** | -0.0333 | -0.0553 | 0.0161 | 0.01276 | -0.06423 | 0.02846 | 0.0362 | 0.02258 | -0.01242 | 0.00129 | -0.02436 | -0.02575 | 0.57369 | - |  |
| **A3** | 0.0023 | -0.01196 | -0.00276 | 0.01525 | -0.02797 | -0.04701 | -0.01181 | 0.02616 | 0.01609 | -0.0281 | -0.00598 | -0.02223 | 0.00244 | 0.08562 | - |

*Note. This matrix shows the correlation residuals (observed correlation – reproduced correlation) of the 3-factor EFA model. Residuals close to 0 indicate a satisfactory local fit. Residuals greater than |0.10| may suggest local areas of misfit. The complete matrix was calculated based on 306 participants (G1). The complete residuals are provided to ensure full methodological transparency. All values are rounded to 5 decimal places for readability*

***Table C2.*** Pattern matrix, commonalities and uniquenesses for the 3-factor EFA model (promax rotation).

| **Item** | **F1** | **F2** | **F3** | **h^2^** | **u^2^** | **Main factor** |
| --- | --- | --- | --- | --- | --- | --- |
| **P3** | **0.55** | 0.22 | 0.15 | 0.65 | 0.35 | F1 |
| **E1** | **0.51** | -0.10 | 0.26 | 0.41 | 0.59 | F1 |
| **E2** | **0.62** | 0.05 | 0.15 | 0.56 | 0.44 | F1 |
| **M1** | **0.85** | 0.12 | -0.17 | 0.71 | 0.29 | F1 |
| **M2** | **0.97** | 0.16 | -0.45 | 0.82 | 0.18 | F1 |
| **M3** | **0.87** | 0.14 | -0.21 | 0.76 | 0.24 | F1 |
| **A1** | **0.78** | -0.08 | 0.01 | 0.54 | 0.46 | F1 |
| **A2** | **0.60** | -0.09 | 0.16 | 0.43 | 0.57 | F1 |
| **A3** | **0.52** | -0.13 | 0.10 | 0.26 | 0.74 | F1 |
| **R1** | -0.14 | **0.80** | 0.02 | 0.53 | 0.47 | F2 |
| **R2** | 0.05 | **0.80** | 0.02 | 0.71 | 0.29 | F2 |
| **R3** | 0.15 | **0.59** | 0.09 | 0.54 | 0.46 | F2 |
| **P1** | **0.36** | 0.25 | **0.36** | 0.66 | 0.34 | F3 |
| **P2** | **0.41** | 0.11 | **0.43** | 0.66 | 0.34 | F3 |
| **E3** | -0.11 | 0.02 | **0.48** | 0.19 | 0.81 | F3 |

*Note. Factor loadings from exploratory factor analysis (method: principal axis factoring, oblique promax rotation, 3 factors). The values reflect the standardized coefficients of the pattern matrix. Communalities (h²) and uniquenesses (u²) are derived from the model. The principal factor corresponds to the highest absolute saturation of the three factors. All values are rounded to two decimal places. Bold values indicate the highest factor loading(s) for each item. Items with similar loadings across multiple factors (e.g., P1, P2) suggest potential cross-loadings.*

**Appendix C3**. Results of parallel analysis (G1, 15 PERMA items)

The parallel analysis was performed with 100 iterations, principal axis factoring (PAF) extraction, on the correlation matrix of 306 participants (group G1). The graph compares the observed eigenvalues with those from random matrices. The retention threshold corresponds to the point where the simulated eigenvalues exceed the observed values. Parallel analysis indicates that a three-factor model should be retained (“Parallel analysis suggests that the number of factors = 3”). Beyond the third factor, the observed eigenvalues fall below those of the random matrices, which does not justify the presence of a fourth or fifth factor in a strict EFA.

***Figure C3.1.*** Scree plot and parallel analysis

*Note. The blue line represents the eigenvalues derived from the observed data. The dashed reference curve corresponds to eigenvalues obtained from simulated and resampled datasets. These two reference curves were nearly identical and therefore overlapped visually. The number of factors was determined by retaining factors for which observed eigenvalues exceeded those from the simulated data, in accordance with Horn (1965).*

***Table C4.*** Phi matrix of interfactor correlations (promax rotation).

|  | **F1** | **F2** | **F3** |
| --- | --- | --- | --- |
| F1 | 1.00 | 0.65 | 0.58 |
| F2 | 0.65 | 1.00 | 0.36 |
| F3 | 0.58 | 0.36 | 1.00 |

***Note****. Phi matrix resulting from an oblique rotation (promax), indicating latent correlations between extracted factors. Moderate to strong positive correlations suggest a multidimensional but interdependent structure, justifying the use of an oblique rotation and the possibility of subsequently testing a hierarchical model or a general factor in confirmatory factor analysis.*

**Appendix D.**

***Table D1.*** Standardized factorial loads and coefficients of determination (R²)

| **Factor** | **Item** | **Standardized load** | **R^2^** |
| --- | --- | --- | --- |
| P | P1 | 0.76 | 0.57 |
| P | P2 | 0.85 | 0.72 |
| P | P3 | 0.96 | 0.92 |
| E | E1 | 0.5 | 0.25 |
| E | E2 | 0.73 | 0.54 |
| E | E3 | 0.3 | 0.09 |
| R | R1 | 0.57 | 0.32 |
| R | R2 | 0.75 | 0.57 |
| R | R3 | 0.9 | 0.81 |
| M | M1 | 0.88 | 0.77 |
| M | M2 | 0.84 | 0.71 |
| M | M3 | 0.93 | 0.87 |
| A | A1 | 0.78 | 0.61 |
| A | A2 | 0.64 | 0.41 |
| A | A3 | 0.48 | 0.23 |
| N1 | 5.32 | 2.49 | 0.00 |
| N2 | 4.21 | 2.14 | 0.00 |
| N3 | 4.38 | 2.28 | 0.00 |
| H1 | 7.08 | 1.85 | 0.00 |
| H2 | 6.03 | 2.45 | 0.00 |
| H3 | 6.90 | 2.10 | 0.00 |
| Lon | 3.52 | 2.94 | 0.00 |
| Hap | 6.94 | 2.13 | 0.00 |

***Note.*** Factor loadings correspond to standardized estimates (λ). R² coefficients indicate the proportion of variance explained for each item by its latent factor.

***Table D2.*** Inter-factor correlations (Phi matrix)

|  | **P** | **E** | **R** | **M** | **A** |
| --- | --- | --- | --- | --- | --- |
| P | 1.00 |  |  |  |  |
| E | 0.81 | 1.00 |  |  |  |
| R | 0.78 | 0.67 | 1.00 |  |  |
| M | 0.85 | 0.83 | 0.78 | 1.00 |  |
| A | 0.75 | 0.81 | 0.62 | 0.83 | 1.00 |

***Note****. Moderate to high positive latent correlations support the hypothesis of an interdependent multidimensional structure.*

***Table D3.*** Complete matrix of standardized residues - CFA 5-factor correlated model (WLSMV, G2)

|  | **P1** | **P2** | **P3** | **E1** | **E2** | **E3** | **R1** | **R2** | **R3** | **M1** | **M2** | **M3** | **A1** | **A2** | **A3** |
| --- | --- | --- | --- | --- | --- | --- | --- | --- | --- | --- | --- | --- | --- | --- | --- |
| **P1** | - |  |  |  |  |  |  |  |  |  |  |  |  |  |  |
| **P2** | 7.46 | - |  |  |  |  |  |  |  |  |  |  |  |  |  |
| **P3** | -1.79 | -6.13 | - |  |  |  |  |  |  |  |  |  |  |  |  |
| **E1** | 0.12 | -1.11 | -3.59 | - |  |  |  |  |  |  |  |  |  |  |  |
| **E2** | 3.06 | 2.35 | -2.96 | -3.98 | - |  |  |  |  |  |  |  |  |  |  |
| **E3** | 0.63 | 0.8 | 0.39 | 0.9 | 3.02 | - |  |  |  |  |  |  |  |  |  |
| **R1** | -3.3 | -2.4 | -0.98 | -2.03 | 4.28 | -1.62 | - |  |  |  |  |  |  |  |  |
| **R2** | -3.03 | -2.57 | 1.01 | -0.72 | -1.23 | 0.14 | 2.49 | - |  |  |  |  |  |  |  |
| **R3** | -0.78 | -1.92 | 6.29 | -1.69 | 0.66 | 0.5 | -0.73 | -1.1 | - |  |  |  |  |  |  |
| **M1** | -2.8 | -1.26 | 0.84 | 1.02 | -1.49 | -1.67 | 0.59 | -0.89 | -1.85 | - |  |  |  |  |  |
| **M2** | -2.75 | 0.3 | -0.18 | 2.57 | 2.12 | -0.86 | 2.59 | 0.09 | -3.1 | 2.92 | - |  |  |  |  |
| **M3** | -2.96 | -0.57 | 5.02 | -0.85 | -1.32 | -1.98 | 0.44 | 4.87 | -0.66 | -0.68 | -2.33 | - |  |  |  |
| **A1** | 1.36 | 0.3 | -0.65 | 3.13 | -1.68 | -1.66 | 0.44 | -1.84 | 0.99 | 0.43 | -0.58 | -1.43 | - |  |  |
| **A2** | -2.18 | 1.26 | 0.87 | 1.89 | -3.94 | -2.2 | -0.96 | -0.07 | -0.31 | 1.95 | 0.72 | -2.12 | 3.06 | - |  |
| **A3** | -3.36 | 1.98 | -1.78 | 2.13 | 0.28 | 1.67 | -1.22 | 3.11 | 0.21 | 0.73 | -0.73 | 2.77 | -2.39 | -1.53 | - |

***Note****. The table shows the standardized residuals from the five-factor correlated CFA model. Several residuals exceed the conventional threshold of |2.58|, indicating localized areas of misfit. However, these residuals were not systematic and did not cluster around specific items or factors, suggesting that they reflect isolated discrepancies rather than an overall misfit of the model. The overall fit indices of the model remained excellent, confirming the appropriateness of the factor structure chosen.*

***Table D4.*** Sensitivity analysis of the PERMA-Profiler five-factor correlated model (CFA, MLR estimator, G2)

|  | **5 correlated factors** |
| --- | --- |
| χ^2^ (df) | 224.63 (80) |
| CFI | 0.943 |
| TLI | 0.925 |
| RMSEA [90% CI] | 0.077 [0.065-0.089] |
| SRMR | 0.044 |

*Note. The items were treated here as continuous variables, with a robust estimation (Maximum Likelihood Robust, MLR). This analysis aims to test the stability of the conclusions regardless of the choice of estimator. Although the fit indices are slightly less favorable than with WLSMV, the overall profile remains compatible with an acceptable fit, confirming the robustness of the factor structure chosen.*

**Appendix E.**

**Table E1.** Detailed correlations between PERMA scores and external variables (with confidence intervals)

| **Score PERMA** | **Criterion** | **n** | **r [95% CI]** | **p** |
| --- | --- | --- | --- | --- |
| PERMA Overall Well-Being | Flourishing (FS total) | 292 | 0.84 [0.80, 0.87] | < .001 |
| PERMA Overall Well-Being | Anxiety (HADS_A) | 296 | -0.44 [-0.53, -0.35] | < .001 |
| PERMA Overall Well-Being | Depression (HADS_D) | 296 | -0.67 [-0.73, -0.60] | < .001 |
| PERMA Overall Well-Being | Negative Emotions | 306 | -0.49 [-0.57,-0.40] | < .001 |
| PERMA Overall Well-Being | Loneliness | 306 | -0.50 [-0.58, -0.41] | < .001 |
| PERMA Overall Well-Being | Happiness | 306 | 0.87 [0.84, 0.89] | < .001 |
| PERMA Overall Well-Being | Health | 306 | 0.49 [0.40, 0.57] | < .001 |
| Positive Emotion (P) | Flourishing (FS total) | 292 | 0.75 [0.69, 0.79] | < .001 |
| Positive Emotion (P) | Anxiety (HADS_A) | 296 | -0.51 [-0.59, -0.42] | < .001 |
| Positive Emotion (P) | Depression (HADS_D) | 296 | -0.66 [-0.72, -0.59] | < .001 |
| Positive Emotion (P) | Negative Emotions | 306 | -0.59 [-0.66,-0.51] | < .001 |
| Positive Emotion (P) | Loneliness | 306 | -0.49 [-0.57, -0.40] | < .001 |
| Positive Emotion (P) | Happiness | 306 | 0.86 [0.82, 0.88] | < .001 |
| Positive Emotion (P) | Health | 306 | 0.46 [0.37, 0.54] | < .001 |
| Engagement (E) | Flourishing (FS total) | 292 | 0.51 [0.42, 0.59] | < .001 |
| Engagement (E) | Anxiety (HADS_A) | 296 | -0.17 [-0.28, -0.06] | 0.003 |
| Engagement (E) | Depression (HADS_D) | 296 | -0.39 [-0.48, -0.28] | < .001 |
| Engagement (E) | Negative Emotions | 306 | -0.22 [-0.33, -0.11] | < .001 |
| Engagement (E) | Loneliness | 306 | -0.20 [-0.30, -0.09] | < .001 |
| Engagement (E) | Happiness | 306 | 0.55 [0.46, 0.62] | < .001 |
| Engagement (E) | Health | 306 | 0.28 [0.17, 0.38] | < .001 |
| Relationships (R) | Flourishing (FS total) | 292 | 0.61 [0.53, 0.68] | < .001 |
| Relationships (R) | Anxiety (HADS_A) | 296 | -0.30 [-0.40, -0.19] | < .001 |
| Relationships (R) | Depression (HADS_D) | 296 | -0.53 [-0.61, -0.45] | < .001 |
| Relationships (R) | Negative Emotions | 306 | -0.31 [-0.41, -0.21] | < .001 |
| Relationships (R) | Loneliness | 306 | -0.49 [-0.57, -0.40] | < .001 |
| Relationships (R) | Happiness | 306 | 0.67 [0.60, 0.73] | < .001 |
| Relationships (R) | Health | 306 | 0.41 [0.32, 0.50] | < .001 |
| Meaning (M) | Flourishing (FS total) | 292 | 0.82 [0.78, 0.85] | < .001 |
| Meaning (M) | Anxiety (HADS_A) | 296 | -0.38 [-0.47, -0.28] | < .001 |
| Meaning (M) | Depression (HADS_D) | 296 | -0.56 [-0.64, -0.48] | < .001 |
| Meaning (M) | Negative Emotions | 306 | -0.41 [-0.50, -0.31] | < .001 |
| Meaning (M) | Loneliness | 306 | -0.43 [-0.51, -0.33] | < .001 |
| Meaning (M) | Happiness | 306 | 0.75 [0.70, 0.80] | < .001 |
| Meaning (M) | Health | 306 | 0.42 [0.32, 0.50] | < .001 |
| Accomplishment (A) | Flourishing (FS total) | 292 | 0.69 [0.62, 0.74] | < .001 |
| Accomplishment (A) | Anxiety (HADS_A) | 296 | -0.38 [-0.47, -0.27] | < .001 |
| Accomplishment (A) | Depression (HADS_D) | 296 | -0.53 [-0.61, -0.44] | < .001 |
| Accomplishment (A) | Negative Emotions | 306 | -0.42 [-0.51, -0.32] | < .001 |
| Accomplishment (A) | Loneliness | 306 | -0.35 [-0.45, -0.25] | < .001 |
| Accomplishment (A) | Happiness | 306 | 0.55 [0.46, 0.62] | < .001 |
| Accomplishment (A) | Health | 306 | 0.38 [0.28, 0.47] | < .001 |

**Appendix F.**

**Table F1.** Test–retest reliability of the PERMA-Profiler total score

| **Sample** | **N** | **r (T1-T2)** | **[95% CI]** |
| --- | --- | --- | --- |
| Full matched sample | 179 | .86 | [.82 ; .90] |
| G1 subsample | 89 | .78 | [.68 ; .85] |
| G2 subsample | 90 | .92 | [.88 ; .95] |

*Note. r = Pearson correlation coefficient between T1 and T2. CI = confidence interval. G1 and G2 correspond to the exploratory and confirmatory subsamples defined in the main analyses.*
